# PVDF Composite Membranes with Hydrophobically-Capped CuONPs for Direct-Contact Membrane Distillation

**DOI:** 10.3390/nano11061497

**Published:** 2021-06-05

**Authors:** César Saldías, Claudio A. Terraza, Angel Leiva, Joachim Koschikowski, Daniel Winter, Alain Tundidor-Camba, Rudy Martin-Trasanco

**Affiliations:** 1 Department of Physical Chemistry, Faculty of Chemistry and of Pharmacy, Pontificia Universidad Católica de Chile, P.O. Box 306, Post 22, Santiago 7820436, Chile; casaldia@uc.cl (C.S.); aleivac@uc.cl (A.L.); 2 Research Laboratory for Organic Polymers (RLOP), Faculty of Chemistry and of Pharmacy, Pontificia Universidad Católica de Chile, P.O. Box 306, Post 22, Santiago 7820436, Chile; cterraza@uc.cl; 3 UC Energy Research Center, Pontificia Universidad Católica de Chile, P.O. Box 306, Post 22, Santiago 7820436, Chile; 4 Fraunhofer Institute for Solar Energy Systems (ISE), 79110 Freiburg, Germany; Joachim.koschikowski@ise.fraunhofer.de (J.K.); daniel.winter@ise.fraunhofer.de (D.W.); 5 Departamento de Química, Universidad Tecnológica Metropolitana, Las Palmeras 3360, Santiago 8940577, Chile

**Keywords:** composite membranes, hydrophobically-capped CuONPs, membrane distillation, fluorinated alkylthiol capping agents

## Abstract

Water scarcity is an imminent problem that humanity is beginning to attempt to solve. Among the several technologies that have been developed to mitigate water scarcity, membrane distillation is of particular note. In the present work, CuO nanoparticles capped with 1-octanethiol (CuONPs@CH) or 1H,1H,2H,2H-perfluorodecanethiol (CuONPs@CF) are prepared. The nanoparticles are characterized by FT-IR and TGA methods. Two weight losses are observed in both cases, with the decomposition of the organic fragments beginning at 158 °C and 230 °C for CuONPs@CF and CuONPs@CH, respectively. Flat sheet PVDF composite membranes containing nanoparticles are prepared by the casting solution method using nanoparticle concentrations that ranged between 2–20% with a non-woven polyester fabric as support. The obtained membranes showed a thickness of 240 ± 40 μm. According to water contact angle (87° for CuONPs@CH and 95° for CuONPs@CF, both at 10% w.t) and roughness (12 pixel for CuONPs@CH and 14 pixels for CuONPs@CF, both at 10% w.t) determinations, the hydrophobicity of membranes changed due to a decrease in surface energy, while, for naked CuONPs, the roughness factor represents the main role. Membranes prepared with capped nanoparticles showed similar porosity (60–64%). SEM micrographs show asymmetric porous membranes with a 200-nm surface pore diameter. The largest finger-like pores in the membranes prepared with CuONPs, CuONPs@CH and CuONPs@CF had values of 63 ± 10 μm, 32 ± 8 μm, and 45 ± 10 μm, respectively. These membranes were submitted to a direct contact membrane distillation module and flux values of 1.8, 2.7, and 3.9 kg(m^2^·h)^−1^ at ΔT = 30 °C were obtained for the CuONPs, CuONPs@CH, and CuONPs@CF, respectively. The membranes showed 100% salt rejection during the testing time (240 min).

## 1. Introduction

In the last 100 years, global water demand has been increasing at an annual rate of 1.8%. This increase has been triggered by population growth, industrialization, and climate change. At present, water demands are becoming a primary source of stress for humans [1,2]. Nowadays, 52% of the global population lives in an area with water scarcity at least one month each year [3]. Unfortunately, of the 3% of the water in the hydrosphere that is fresh water, only 30% is potable, and illegal human activities (such as chemical discharge) may pollute potable water [3,4,5,6]. Approximately 97% of the global water reserve corresponds to oceans, which additionally are ubiquitous. The desalination of seawater seems to be a very plausible option to meet growing water demands [5].

Thermal-based (multi-stage flash distillation and multiple-effect distillation) and membrane-based processes (reverse osmosis (RO), nanofiltration (NF) and membrane distillation (MD)) are the two main technologies used for the desalinization of seawater [7]. Due to the higher energy consumption of thermal-based technologies over membrane-based technologies, interest in the latter has been increasing over the last three decades. Accounting for membrane-based technologies, although 69% of the worldwide desalinated water is produced via seawater reverse osmosis (SWRO), this technique is still considered to be a high energy consumption technology with a negative environmental impact associated with the emission of greenhouse gases [8,9].

Membrane distillation (MD) describes a non-isothermal water purification technology in which a porous hydrophobic membrane is used. The membrane separates a hot feed water solution from a cold permeated solution. The difference in temperature between both sides creates a vapor pressure gradient, which is the driving force of the process. Due to the hydrophobic nature of the membrane, only water vapor passes through it, and therefore it theoretically rejects 100% of salts and other non-volatile solutes on the feed side. Thus, fresh water condenses on the permeated side [5,10].

Although MD is entails higher energy consumption than other membrane-based process (thermal energy is required to heat the water feed), this consumption can be reduced using renewable and low-grade energies [11,12,13,14,15]. Additionally, operational performance at high salinity conditions and small space requirements are some of the features that distinguish MD over other techniques like RO. From the variety of MD setups, the most technologically simple and least energy demanding technique is direct contact membrane distillation (DCMD) [5,16].

An outstanding MD membrane should have a high liquid entry pressure (LEP), high mass transference, chemical and fouling resistance (useful for long-term use), high mechanical strength, and low thermal conductivity. Changes in the membrane material and its structure, composition, and surface morphology are some of the commonly tuned parameters to achieve these features. Among them, increasing the LEP and flux and lowering the fouling rate are common targets in designing more efficient membranes for MD [5].

From economic, energy, and processing points of view, polymeric membranes are preferred over ceramic-based membranes. Due to the high hydrophobicity of fluoropolymers such as polytetrafluorethylene (PTFE) and polyvinylidene fluoride (PVDF), fluoropolymers are preferred for preparing polymeric MD membranes. Nevertheless, since PVDF is soluble in most common aprotic polar solvents, this polymer widens the possibilities of membrane composite innovation.

Research works have been carried out to improve the performance of PVDF in MD. Hou et al. fabricated a nanofibrous PVDF composite membranes by an electrospinning technique to improve the mechanical properties of the electrospun nanofibrous membranes [17]. Bonaccorso et al. enriched a PVDF membrane with a few layers of graphene. The modification increased the flux and consequently rejected 100% of salts [18]. Another approach in membrane innovation is the addition of metal, metal oxide, or carbon derivate nanoparticles [19,20,21,22,23,24].

The most common method to prepare PVDF porous flat sheets or hollow fiber membranes is non-solvent induced phase separation (NIPS). The hydrophilic nanoparticle concentrations in membranes prepared via NIPS should not exceed 2 wt %, since at a higher concentration they tend to form aggregates [5]. Nevertheless, it has been reported that this concentration can be increased to 5 wt % by using hydrophobic nanoparticles [25].

At the aforementioned concentration, hydrophobic nanoparticles do not have a great effect on the surface hydrophobicity (i.e., the water contact angle (WCA) could increase to approximately 10°). Nevertheless, increasing pore size, reducing pore wetting, and decreasing temperature polarization are some of the effects of preparing PVDF composite membranes using hydrophobically surface-modified nanoparticles. All these features contribute to increasing the flux [26,27].

Another important feature to consider in the preparation of membranes is resistance to fouling. The fouling of membranes (biological, organic, and inorganic) affects their long-term usability and efficiency in MD processes, and thus increases the operational cost. Designing efficient hydrophobic membranes with a high resistance to any class of fouling is a challenge, as an attempt to mitigate a given class could enhance any other class of fouling [5,28]. Depending on the application, it is possible to modify the hydrophobicity of PVDF-based membranes. For instance, in micro- and ultra-filtration processes, the membrane needs to be more hydrophilic, which can be achieved by incorporating molecules with polar groups or polar fragments on the surface of the membrane [29,30,31,32,33]; however, in MD, the performance of the membrane increases by increasing the hydrophobicity of the active layer of the membrane. In the case of biofouling, composite membranes with antimicrobial agents such as TiO_2_NPs, CuONPs, AgNPs, graphene oxide, and ZnONPs have been prepared by incorporating these nanoparticles into the PVDF casting solution [19,24,27,34].

The bactericidal and antimicrobial activities of CuONPs have been well established [35,36]. Nevertheless, few works have reported the use of these nanoparticles for preparing MD membranes [34,37,38,39]. Recently, we prepared CuONP-PVDF composite membranes supported on non-woven polyester fabric (NWPET) at different CuONP concentrations. The nanoparticle concentration affects the crystalline phase, pore distribution, and morphology and surface hydrophobicity [19]. 

In the present work, we report the preparation and characterization of hydrophobic CuONP-PVDF composite membranes supported on non-woven polyester fabric (NWPET) and their performance in water desalinization with a DCMD technique. The CuONP surfaces are modified with *n*-octanethiol (CH) or 1H,1H,2H,2H-perfluorodecanethiol (CF) as hydrophobic capping agents, and the latter is additionally considered to be a superoleophilic substance [40,41,42,43,44]. PVDF composite membranes prepared with CuONPs capped with CH or CF will have larger flux values than those prepared with naked nanoparticles. The novelty of this work lies in the preparation of hydrophobically capped CuONPs for the first time and the resulting effects in preparing PVDF-CuONP composite membranes for membrane distillation.

## 2. Experimental

### 2.1. Materials

Non-woven polyester fabric (NWPET) was purchased from Importadora Dilaco S.A. (Santiago, Chile). Copper oxide nanoparticles (CuONPs, diameter < 50 nm), poly(vinylidene fluoride) (PVDF, average molecular weight of ~180,000 kDa by GPC, average Mn ~71,000, beads or pellets), *N,N*-dimethylformamide, (DMF, ≥99.8%), ethanol (≥99%), *n*-octanethiol (≥98.5%), and 1H,1H,2H,2H-perfluorodecanethiol (97%) were purchased from Sigma-Aldrich (Milwaukee, WI, USA) and were used without further purification.

### 2.2. Methods

#### 2.2.1. Surface Modification of CuONPs

A round bottom flask containing ethanol (400 mL), CuONPs (400 mg) was dispersed by bath sonication for 1 h. *n*-octanethiol or 1H,1H,2H,2H-perfluorodecanethiol (0.4 mL) were added and the dispersion was submitted to sonication for 30 min and then stirred overnight. Capped CuONPs were filtered out and washed with ethanol. The nanoparticles were again re-dispersed in ethanol by sonication and the filtration and washing processes were repeated to remove excess alkylthiols on nanoparticle surfaces. The obtained modified CuONPs (CuONPs@CH or CuONPs@CF) were dried overnight in a vacuum oven at 40 °C.

#### 2.2.2. Preparation of the PVDF Casting Solution Containing CuO Nanoparticles 

A polymer solution (20% *wt*./*v*) was prepared by dissolving an adequate amount of PVDF in DMF while stirring at 100 °C. After dissolving the polymer, the solution was stirred at room temperature for an additional 12 h. PVDF composite membranes were prepared at different nanoparticle/PVDF weight ratios (2, 5, 10, and 20%). A homogeneous dispersion stock of the corresponding naked CuONPs or modified (0.4 g/mL) in DMF was prepared by several sonication and stirring processes. From this, an adequate volume was taken to achieve the desired nanoparticle/PVDF wt % (2, 5, 10, and 20%). The nanoparticle dispersion was dropped into the polymer solution with vigorous stirring and was degassed via the application of a vacuum under sonication.

#### 2.2.3. Preparation of PVDF-CuO Composite Membranes with CuO Nanoparticles 

The PVDF suspension containing the nanoparticles was cast onto NWPET (210 mm × 297 mm) that was previously impregnated with DMF. NWPET was fixed in a hand-made PTFE frame with a thickness of approximately 0.5 mm and was used as template to build the membrane (Figure S1). After waiting for 30 s, the PTFE template with the NWPET and casted film was gently dipped into distilled water at 25 °C as a non-solvent to propitiate the precipitation of the polymer. The membrane was left into the coagulation bath for 24 h. Meanwhile, the liquid was often replaced with distilled water to remove any solvents. Finally, the membrane was left to dry overnight in an oven at 50 °C. A membrane thickness of 240 ± 40 μm was obtained as determined from the cross-section SEM micrographs (Figure S2).

#### 2.2.4. Equipment

##### ATR-FT-IR

Infrared spectra were recorded on a Perkin-Elmer Spectrum-Two spectrometer (PerkinElmer Inc., Waltham, MA, USA) with a coupled Universal Attenuated Total Reflection (UATR) unit. Samples were placed over the diamond, pressed until reaching 30% of the total supported pressure, and scanned in the range from 4000 to 500 cm^−1^ with a resolution of 1 cm^−1^.

##### Thermogravimetric Measurements

Thermogravimetric analysis (TGA) was performed using a TGA/SDTA851 Mettler Toledo thermal analyzer (Greifensee, Switzerland) in an air atmosphere at a heating rate of 10 °C min^−1^. 

#### 2.2.5. Characterization of PVDF-CuO@CH and PVDF-CuO@CF Composite Membranes 

##### Scanning Electron Microscopy

Scanning electron microscopy was performed with a Zeizz model EVO MA 10 electron microscope (Oberkochen, Germany) in order to study the surface and cross-section membrane morphologies. The cross-section SEM micrographs were acquired by fracturing the membranes using liquid nitrogen to freeze them and a surgical scalpel to cut the NWPET. The membranes were coated with gold using a Cressington-108 auto sputter coater (Zeizz, Oberkochen, Germany). The measurements and processes of the obtained SEM micrographs were performed using the free ImageJ (version 1.46 J/Fiji) software package from the National Institute of Health, Bethesda, MD, USA [45].

##### Water Contact Angle Measurements

Water contact angle (WCA) measurements were performed by means of the sessile drop technique using Dataphysics OCA 20 (DataPhysics, Filderstadt, Germany). A syringe connected to a capillary of Teflon with an approximate 2-mm internal diameter was used to place a water drop (10 μL) on the membrane surface. The acquisition of images was carried out by a camera coupled to the equipment and the WCA values were obtained through computational processing of the drop profile via determining tangent lines. At least five measurements were taken at different sites of the membrane and the reported WCA values are averages of these measurements.

##### Surface Roughness Determination 

SurfcharJ, a plugin for the ImageJ software package (version 1.52, Wayne Rasband, Rockville Pike, Bethesda, Maryland) was used to perform local roughness analysis of the SEM micrographs to determine the surface roughness according to the ISO 4287/2000 standard [39,46]. A 32-bit two-dimensional SEM image is converted into a three dimensional image with pixel values ranging from 0–255 and an assigned z-distance to the surface. In the gray scale, lower values (→0) represents lower sector spaces and lighter areas with higher values (→255) correspond to upper zones. The standard deviation of pixel brightness values is a measure of the hill/void-space frequency and can be used as a measure of surface uniformity. Moreover, the higher the standard deviation, the higher the surface roughness. The software affords the following parameters: Ra: arithmetical mean deviation; Rq: root mean square deviation; Rv: lowest valley; and Rp: highest peak. For each sample, a duplicate specimen was analyzed and four different areas were randomly selected in each one.

##### Membrane Porosity

The membrane porosity was determined by the gravimetric method using *n*-butanol as a wetting solvent. The prepared membranes were fully immersed in a sealed flask with a solvent for 24 h to ensure complete pore wetting. The wet membranes were removed from the flask, then superficially dried by gently pressing between two pieces of filter paper and then weighed (*w*_1_). The membranes were weighed again after drying in an oven at 50 °C (*w*_2_). The membrane porosity (*ε*) was calculated as per Equation (1):

(1) $$\varepsilon \left(\%\right)=\frac{\left(w_{1}-w_{2}\right)\cdot \rho_{2}}{\rho_{2}w_{1}+\left(\rho_{1}-\rho_{2}\right)w_{2}}\times 100\;$$

where *ρ*_1_ and *ρ*_2_ are the density of PVDF (1.78 g·cm^−3^) and *n*-butanol (0.810 g·cm^−3^), respectively, at 25 °C. In order to obtain a representative porosity value, the weighing processes were performed under the same conditions with three different samples from the same membrane and the results were finally averaged.

##### Membrane Performance

**Figure 1:**
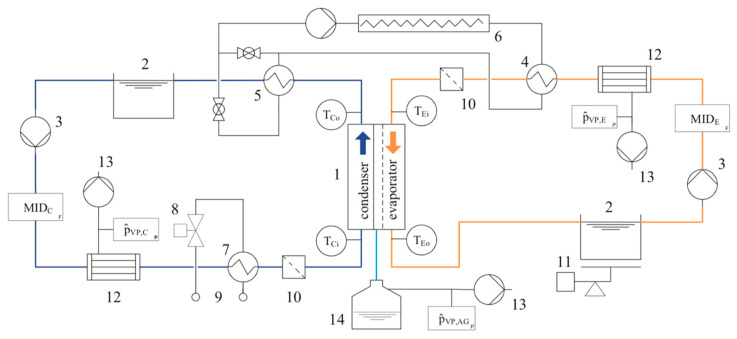
Hydraulic layout of the experimental setup; 1: membrane test cell; 2: feed water tank; 3: feed pump; 4: heat exchanger; 5: auxiliary heat exchanger; 6: electric heater; 7: heat exchanger; 8: motorized valve; 9: laboratory cooling circuit; 10: filter; 11: electronic balance; 12: deaeration module; 13: vacuum pump; 14: permeate tank [47].

The membrane performance was evaluated with a DCMD setup in a membrane distillation unit as schematically represented in Figure 1. The experimental setup consisted of a DCMD cell with 10-L feed and permeation chambers which were weighed during the experiment. Two temperature controllers adjusted the feed and permeation temperatures. Membrane performance was evaluated in triplicate while measuring the flux at three different feed and permeation temperatures (T_f_–T_p_ = 64–56 °C, 64–44 °C, and 80–50 °C). The feed solution consisted of a 0.1 wt % aqueous sodium chloride solution. The flux was measured according to Equation (2):

(2) $$J=\frac{w}{A\cdot t}\;$$

where *J* is flux in kg (m^2^·h)^−1^, *w* (kg), *A* = 0.0375 m^2^, and *t* (h) are the weight of the permeate collected, effective membrane area, and filtration time, respectively. The salt retention was checked by measuring the conductivity. 

## 3. Results and Discussion 

### 3.1. Preparation and Characterization of Hydrophobically Capped CuONPs 

#### 3.1.1. ATR-FT-IR Characterization

**Scheme 1:**
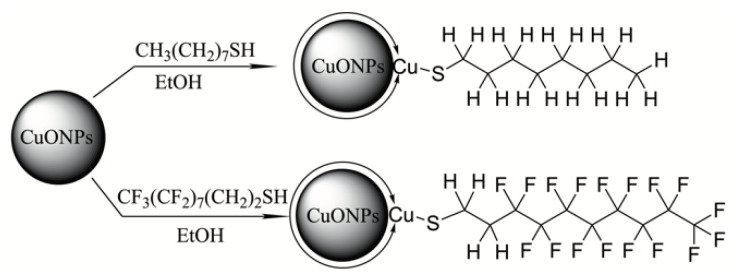
Surface modification of CuONPs with *n*-octanethiol and 1H,1H,2H,2H-perfluorodecanethiol.

According to a previous report, thiol compounds efficiently coordinate with the metals of groups 8 and 11 and their corresponding oxides. This coordination should lead to the breakdown of the S-H bond with the formation of thiolate species [40,41]. Hydrophobically capped CuONPs were prepared by the ligand exchange method using *n*-octanethiol or 1H,1H,2H,2H-perfluorodecanethiol. In these compounds, the thiolate function should coordinate the copper (II) at the nanoparticle surface (Scheme 1). 

**Figure 2:**
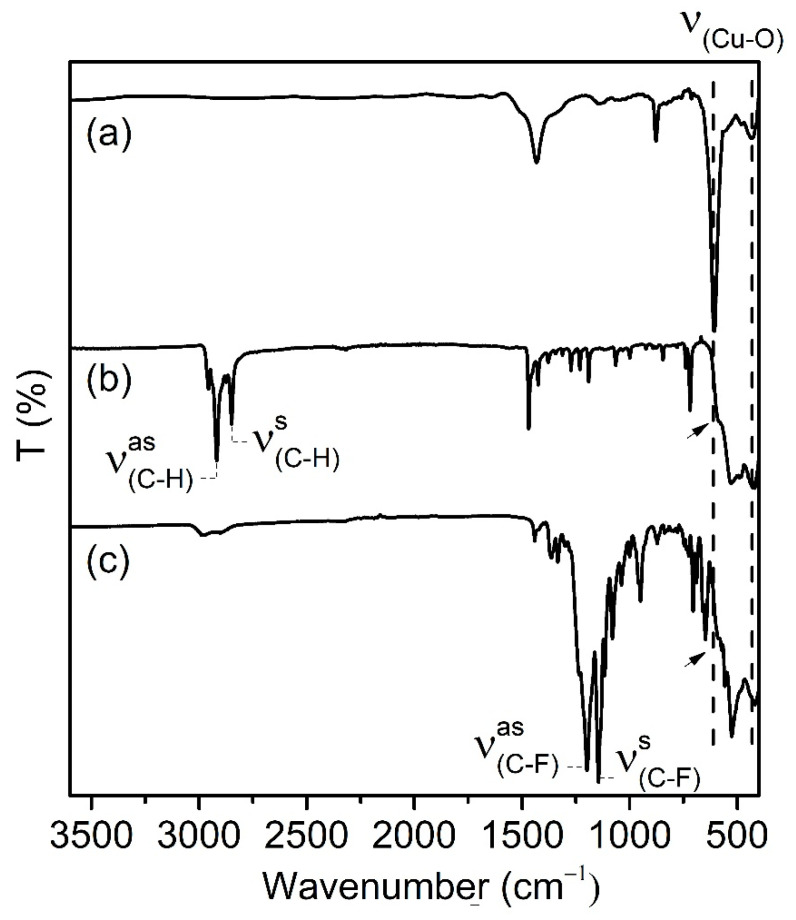
FT-IR spectra of (**a**) CuONPs, (**b**) CuONPs@CH, and (**c**) CuONPs@CF.

In the FT-IR spectrum of CuONPs (Figure 2a), the signals recorded at 429 and 607 cm^−1^ may be assigned to Cu-O stretching along the different crystal planes of CuO as reported elsewhere [48]. The spectrum of the modified nanoparticles with *n*-octanethiol shows the typical pattern of linear alkyl hydrocarbon (Figure 2b). The two peaks at 2849 and 2918 cm^−1^ correspond to symmetric $\left(\nu_{CH}^{s}\right)$ and anti-symmetric $\left(\nu_{CH}^{as}\right)$ stretching in the methylene in alkyl chain, respectively. 

The two signals mentioned before were also recorded at 2902 cm^−1^ and 2988 cm^−1^ in the FT-IR spectra of nanoparticles modified with fluoroalkyl chains which contained two of these methylene groups (Figure 2c). Additionally, two intense bands corresponding to symmetric $\left(\nu_{CF}^{s}\right)$ and anti-symmetric $\left(\nu_{CF}^{as}\right)$ stretching in the difluoromethylene groups were recorded at 1145 and 1198 cm^−1^, respectively. One may notice that in the spectra of the modified nanoparticles, the bands of CuO were also recorded, although the intense band at 607 cm^−1^ appears as a shoulder (marked by arrows). The non-detection of a S-H stretching band between 2500–2700 cm^−1^ (Figure S3) suggest the formation of the thiolate due to the coordination of the copper at the nanoparticle surface.

#### 3.1.2. Thermogravimetry Analysis (TGA)

**Figure 3:**
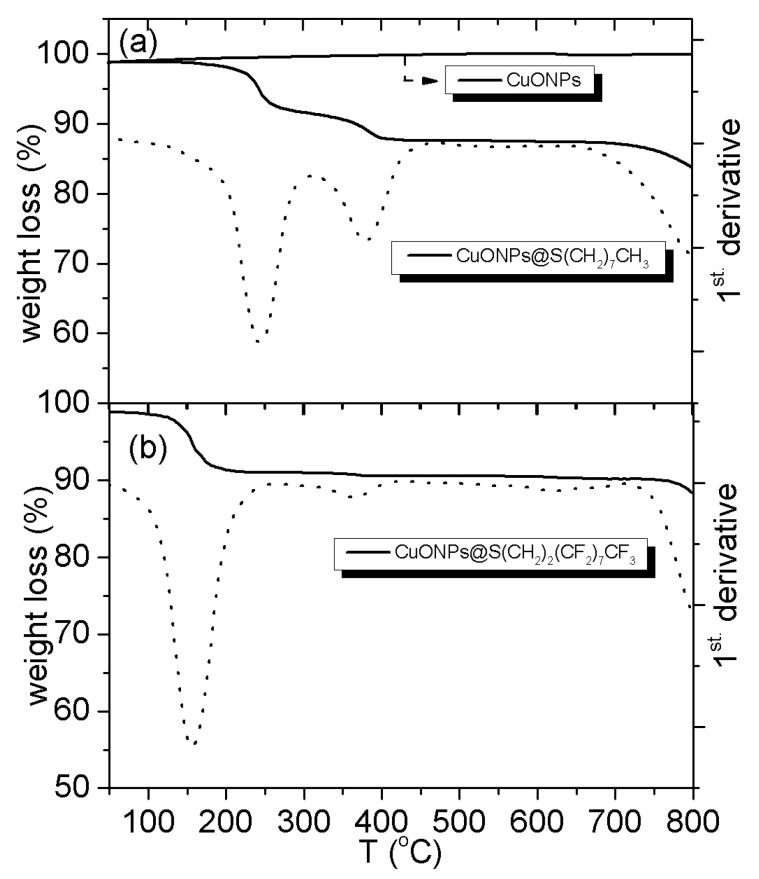
Thermograms and the corresponding differential curves for (**a**) naked CuONPs and CuONPs@CH and (**b**) CuONPs@CF.

The organic weight fraction of a capped nanoparticle can be determined by TGA. The organic component thermally decomposes to provide volatile compounds. The presence of the capping agents on a CuONP surface should be detected by this technique. Consequently, thermograms were recorded for naked and capped CuONPs in the range of 50 to 800 °C (Figure 3). Naked CuONPs did not show weight loss in the temperature range studied, which indicates the absence of any volatile component on CuONP cores (Figure 3a). Nevertheless, in the thermogram of CuONPs@CH, weight losses of 6% and 2% at 246 °C and 378 °C, respectively, were recorded (Figure 3a), where the former showed a higher slope than the latter, suggesting that the removal of 1-octanethiolate species started at 230 °C. One may notice that a discrete weight loss (approximately 2%) can be observed between the first and second steps. This behavior is typical from the gradual decomposition of the alkane tail of capping agent. A second step starts at 360 °C and can be attributed to the decomposition of the residual Cu-S surface species.

Similar to the CuONPs@CH, the thermogram recorded for the CuONPs@CF showed two weight losses. The first step at 158 °C shows a weight loss of 8%. Assuming that same decomposition process as in a CH monolayer occurs and considering that the nanoparticles features were the same in both experiments, we can suggest that the amount of absorbed CF was lower than the CH here. As the theoretical weight ratios of CF and CH are 3.28 from the TGA measurements, we obtained a weight loss ratio of 1.33. A second weight loss was recorded at 356 °C (1.5%), very close to the second weight loss as in CH, which is a result that appears to be due to the same process (i.e., the decomposition of residual Cu-S species). In both thermograms, significant weight loss was observed at temperatures higher than 700 °C, which could be attributed to the total oxidation of the carbonaceous material to CO_2_. 

### 3.2. Membrane Preparation and Characterization

The membranes were prepared by casting a polymer solution containing naked and surface modified CuONPs at 2, 5, 10 and 20 wt % on NWPET. The NWPET was fixed in the hand-made membrane template and the polymer solution was spread on this by using a glass bar. Independent of the concentration and nanoparticle, the membrane thicknesses were 180 ± 40 μm (Figure S3). The NWPET serve as hydrophilic side membrane oriented to the permeation site, allowing water condensation and guaranteeing the necessary mechanical strength of the membrane. The hydrophobic active side, i.e., the PVDF, is in contact with the feed solution and therefore has the main role in the distillation process.

#### 3.2.1. Water Contact Angle, Membrane Roughness and Porosity

**Figure 4:**
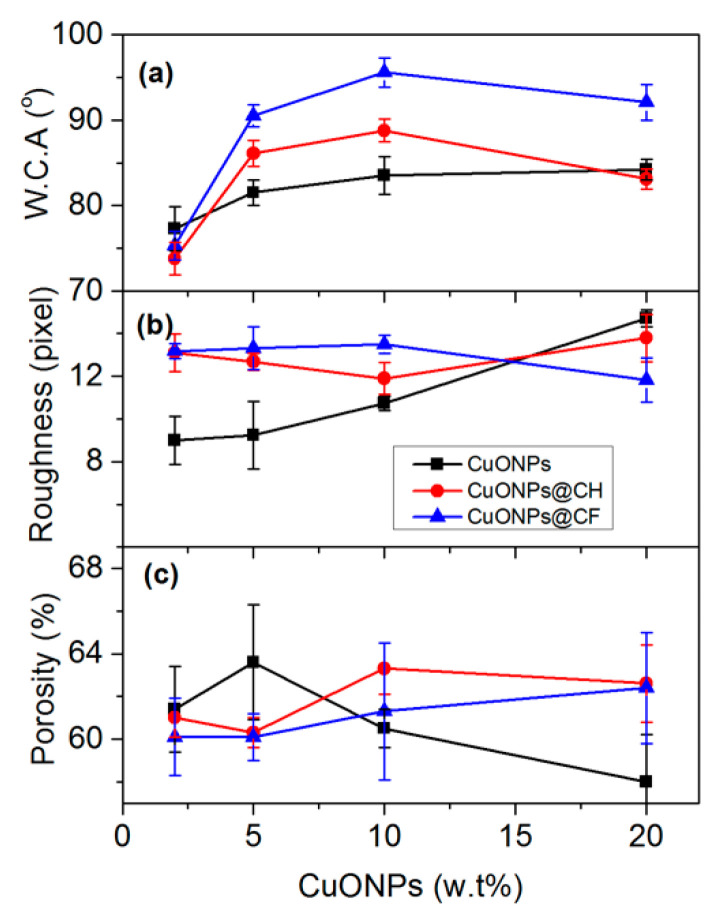
Variation of (**a**) WCA, (**b**) membrane surface roughness, and (**c**) film porosity with varying nanoparticle types and concentrations.

Water contact angle (WCA) measurements were taken to determine the effect of the type and concentration of nanoparticle on the PVDF surface hydrophobicity. Figure 4a shows the WCA values of the prepared membranes at different nanoparticle concentrations. Independent of the type of nanoparticle (either hydrophilic or hydrophobic), at a lower concentration (2%), the WCA values were similar and close for the PVDF (72.8° ± 3.8). At concentrations ranging from 2–10%, an increase in the WCA was observed, although the increase was greater in the case of hydrophobic nanoparticles. Interestingly, at a higher concentration (20%), the WCA decreased for capped nanoparticle but did not for the naked nanoparticles. It is well known that PVDF membranes prepared by the NIPS method, using water as a non-solvent, presents WCA values ranging from 70° to 100° [39,49]. The latter value has been never beaten with direct prepared PVDF membranes unless a post surface treatment has been applied made or by using an alcohol as the non-solvent [22,23,50,51]. We expected to exceed this value with the hydrophobically capped CuONPs, but it never surpassed 100° here.

The water contact angle depends on the surface energy (chemical composition of surface) and roughness. An increase in the WCA can be attributed to either lowering the surface energy or increasing the surface roughness [5]. It is important to note the unexpected results here, i.e., (i) the increases in the WCA with the increases in the concentration of hydrophilic nanoparticles (naked CuONPs) and (ii) the decreases in the WCA with the addition of hydrophobically-capped CuONPs at concentrations beyond 10%. The interplay between a hydrophobic membrane and surface roughness could produce this result.

Membrane roughness (Figure 4b) was determined from corresponding surface SEM micrographs. Surface roughness of membranes with naked CuONPs, increases in the whole range of concentration (2–20%). Naked CuONPs are hydrophilic, and due to their unprotected surface they tend to aggregate when increasing their concentration in order to release excess surface energy (Figure S4a). This aggregation should provoke the growth of bulky aggregates underneath of the surface, which increases the roughness and therefore the WCA.

The roughness values of of membranes containing CuONPs@CH and CuONPs@CF were similar in the lower range of concentration (2–10%), nevertheless, the WCA increased. These results can be expected when considering that an increase in the WCA is due to a lower surface energy. Capped CuONPs interact with PVDF alkyl chains in the casting solution to a higher extent. This fact favors their dispersion in the whole solution and therefore in the membrane upon precipitation. Hydrophobic capped nanoparticles at the surface are responsible for decreasing the surface energy and therefore increasing the hydrophobicity of the membrane. 

At 10%, the CuONPs@CH also started to form aggregates, although to less of an extent than the naked CuONPs (Figure S4b,c). Independent of the type of nanoparticle, aggregates are formed at concentrations higher than 10% and their surface area decreases, which could explain the observed decreases in the WCA. Additionally, at 20%, the aggregation becomes brittle because of the compromised mechanical integrity of the active layer (PVDF). This is a condition that excludes use for membrane distillation.

Ideal membranes should have high porosity in order to lower the thermal conductivity and propitiate high flux, but this should not compromise their mechanical properties. The effects of the types and concentrations of nanoparticles in the membrane porosity (ε) were determined by the wet method and the results are depicted in Figure 4c. 

The porosity of a membrane with naked CuONPs was increased from 60% to 62% by increasing the nanoparticle concentration from 2% to 5%; however, decreases were observed once reaching 58% for 20% of nanoparticles. At this concentration, the aggregation of nanoparticles takes place and the aggregates block the pores.

The porosity of membranes with CuONPs@CH showed a low decrease from 62% to 60% when the concentration increased from 2% to 5%, respectively. Nevertheless, at a concentration of 10%, the porosity increased to 65%. At 20% of nanoparticles, the porosity barely changed with respect to the latter. The membranes prepared with CuONPs@CF showed a slight increases in the whole range of concentration studied. In this membrane, the porosity changed from 60% to 63% by increasing the nanoparticle concentration from 2% to 20%, respectively.

#### 3.2.2. SEM Micrograph Analysis of Membranes

An essential requirement in the preparation of MD membranes is the presence of pores on their surface. The pores located on the surface must not exceed 400 nm in size, otherwise there is a risk that liquid water will penetrate into the membrane. Similarly, the pores near the surface are required to have a finger-like morphology to promote a capillary effect that increases the vapor pressure of the liquid and favors flux into the membrane. The size, distribution, and morphology properties of the pores of the different prepared membranes were studied by means of scanning electron microscopy.

**Figure 5:**
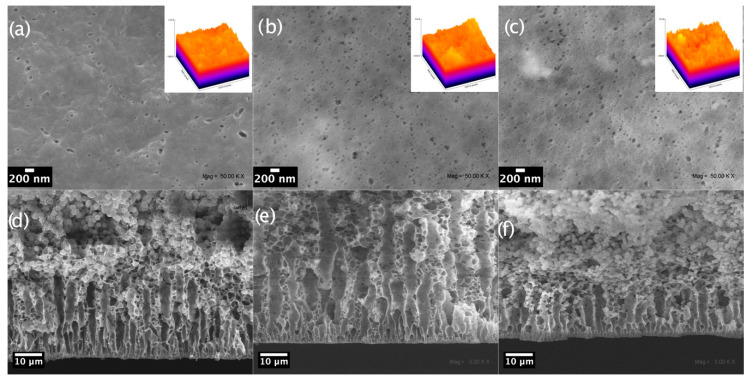
Surface (**a**–**c**) and cross-section (**d**–**f**) SEM micrograph images of PVDF composite membranes prepared at 2% (**a**,**d**), 5% (**b**,**e**), and 10% (**c**,**f**) CuONPs. The insets in the upper row show 3D color surface roughness profiles (x = 768 px; y = 1024 px; z (intensity): −160 to 113)).

**Figure 6:**
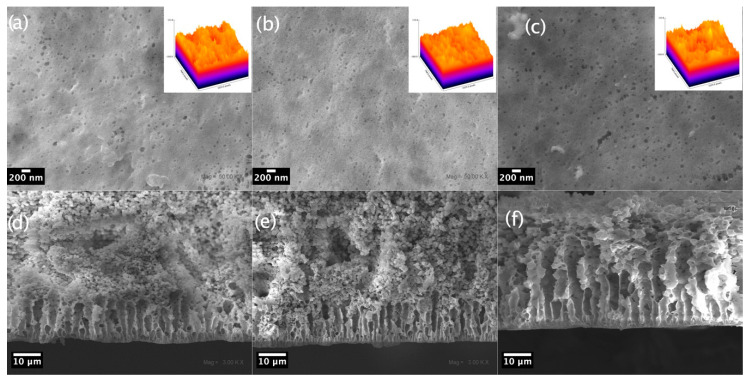
Surface (**a**–**c**) and cross-section (**d**–**f**) SEM micrograph images of PVDF composite membranes prepared at 2% (**a**,**d**), 5% (**b**,**e**), and 10% (**c**,**f**) CuONPs@CH. The insets in the upper row show 3D color surface roughness profiles (x = 768 px; y = 1024 px; z (intensity): −160 to 113)).

**Figure 7:**
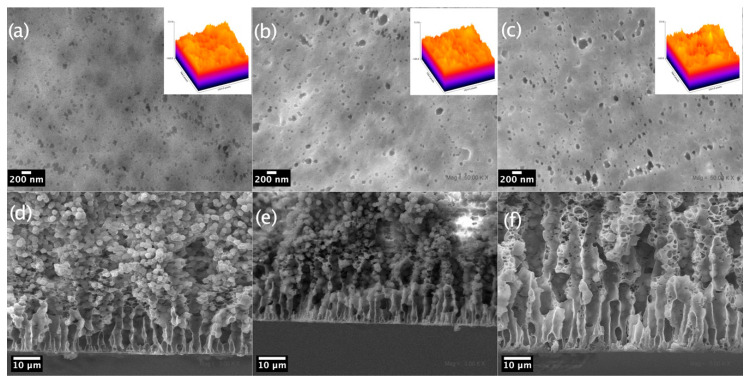
Surface (**a**–**c**) and cross-section (**d**–**f**) SEM micrograph images of PVDF composite membranes prepared at 2% (**a**,**d**), 5% (**b**,**e**), and 10% (**c**,**f**) CuONPs@CF. The insets in the upper row show 3D color surface roughness profiles (x = 768 px; y = 1024 px; z(intensity): −160 to 113)).

Figure 5, Figure 6 and Figure 7 show surface (upper row) and cross-section (lower row) SEM micrographs of membranes prepared at different concentrations with varying CuONP types. As can be seen in the SEM cross-section, all the images show a top surface skin-layer supported by a finger-like porous layer. A sponge-like layer is beneath the finger-like layer. These asymmetric features are typical for membranes prepared by a non-induced phase separation (NIPS) method.

The surface SEM micrographs of the membrane containing naked CuONPs (Figure 5) shows pores on it, and except for the membrane with the lowest CuONP concentration (5%), the two others show similar surface pore sizes (120 ± 30 nm). The highest surface pore density was obtained for the membrane prepared with 5% CuONPs. 

Similarly, all cross-section micrographs show finger-like pore morphologies that extend from the top-skin layer of membrane to the inner layer, and more inner pores with a sponge-like morphology. The finger-like pore layer length increased from 32 ± 8 µm to 63 ± 10 µm at CuONP concentrations of 2% and 5%, respectively. Beyond this concentration, the pore length decreases to 21 ± 7 µm (CuONPs at 10%). 

Figure 6 shows SEM micrographs of membranes prepared with CuONPs@CH. As depicted in Figure 5a–c, the surface pore density increased with an increase in nanoparticle concentration. The highest surface pore density was reached in the membrane prepared with 10% nanoparticles with an average pore diameter of 150 ± 20 nm. Regarding the cross-section SEM micrographs (Figure 6d–f), at concentrations of 2% and 5% of CuONPs@CH, the finger-like pores showed similar lengths (13 ± 3 µm and 12 ± 5 µm) with well-defined boundaries between the finger-like and sponge-like layer; however, at 10% CuONPs@CH (Figure 6f), the pores were approximately twice as large than those at a lower concentration (32 ± 5 µm). 

The surface SEM micrographs of membranes prepared with CuONPs@CF (Figure 7) show the larger surface pores sizes (200 ± 23 nm) when compared to those prepared with CuONPs and CuONPs@CH. The membranes prepared at 5% and 10%, although presenting larger surface pores than that with 2%, also show higher dispersion for size (80 nm). When increasing the nanoparticle concentration, the cross-section SEM micrographs (Figure 6d–f) showed an increase in the finger-like pore length with values of 16 ± 6 µm, 24 ± 8 µm and 45 ± 8 µm at concentrations of 2%, 5%, and 10% for CuONPs@CF, respectively.

The results can be interpreted by considering the mechanism of pore formation via NIPS proposed by Smolder et al. [52]. This mechanism state that the formation of an asymmetric porous membrane results from the separation (demixing) of a polymer-rich phase (solid phase of the membrane) from a polymer-poor phase which forms pores. The demixing process obeys the thermodynamic stability of the casting solution and the kinetics of solvent/non-solvent exchange [53,54].

Summarizing our results:

I. In membranes containing CuONPs, the finger-like pore lengths increased when the nanoparticle concentration increased from 2% to 5% but decreased at 10%.
II. In membranes containing CuONPs@CH, finger-like pore lengths were similar at the two lower nanoparticle concentrations but larger at 10%.
III. In membranes containing CuONPs@CF, finger-like pore lengths increased when increasing the nanoparticle concentration.

Accordingly, the proposed demixing mechanisms are the following:

(a) A casting solution with hydrophilic nanoparticles (naked CuONPs) draws non-solvent molecules (water) to a deeper level into the polymer solution and at a higher rate when the concentration increases from 2% to 5%. The faster the demixing, the larger the finger-like pore layer. At a concentration of 10%, the casting solution increases in viscosity and delayed demixing takes place. Under this regime, a decrease in the finger-like layer thickness occurs as observed here.
(b) A casting solution with hydrophobically capped nanoparticles (CuONPs@CH and CuONPs@CF) has low affinity for the used solvent (DMF) but high affinity for the polymer backbone (-(CF_2_-CH_2_)-). Therefore, the casting solution becomes more thermodynamically unstable when increasing the nanoparticle concentration. The more unstable the solution, the higher the demixing rate and the larger the formed finger-like pores [55].

#### 3.2.3. Membranes Performance Measurements

**Figure 8:**
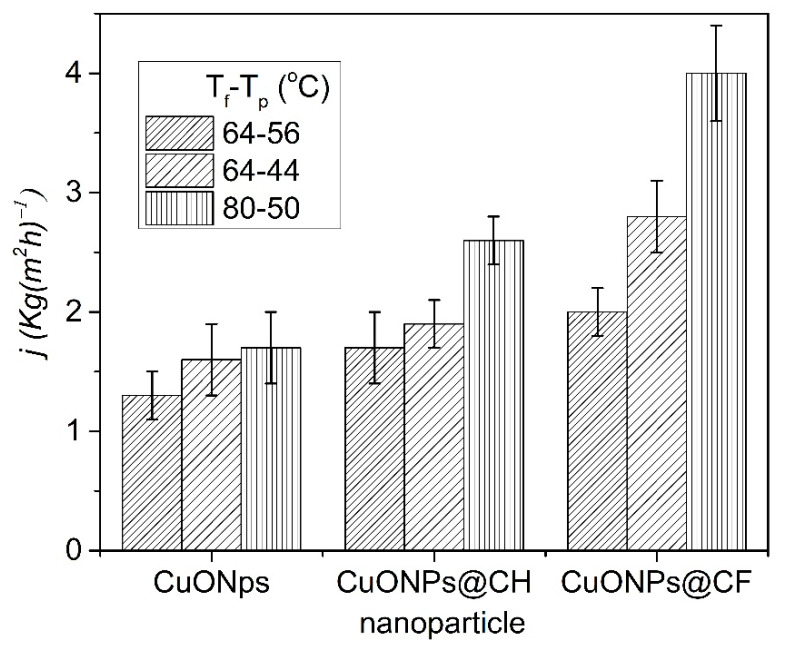
Performance of membranes prepared with 5% CuONPs and 10% CuONPs@CH and CuONPs@CF.

According to the cross-section SEM micrographs and porosity results, for naked CuONPs, the membrane prepared at 5% had higher porosity and the largest finger-like pores. Therefore, it was selected to study its performance for membrane distillation. Considering that for CuONPs@CH and CuONPs@CF, neither porosity nor WCA values have a significant difference between them, and as such the selection criterion was made only according to the finger-like pore length. Therefore, the preparations with 10% nanoparticles were selected to check their water distillation ability. Figure 8 shows the flux achieved for each selected membrane at three different ΔT = T_feed_ − T_permeated_ temperature values (64–56 °C, 64–44 °C, and 80–50 °C).

As expected, the flux of each membrane increased by increasing the difference between the feed and permeation temperatures. The membrane containing naked CuONPs showed the lowest increase with temperature (1.1 time), while the membranes with the CuONPs@CH and CuONPs@CF increased their fluxes by 1.5 and 2.3 times at the highest ΔT, respectively.

This low permeation of membrane with CuONPs is in agreement with the porosity and EDS results. The porosity of this membrane increased from 61% to 63% and then decreased to 60% with an increasing concentration from 2, 5 and 10% of CuONPs, respectively. In the cross-section EDS mapping micrograph, several aggregates which correspond to CuONPs can be seen (Figure S4). These aggregates increased in size at the highest concentration (10%). We suggest that CuONP aggregates not only avoid the passage of vapor, but also allow water condensation inside the membrane.

At the same ΔT, the membrane containing CuONPs@CF shows higher permeation fluxes than CuONPs@CH. Additionally, the increases in flux in the membrane prepared with CuONPs@CF with the increases in temperature is greater than in the membrane with CuONPs@CH. Considering the similarity in the porosity, thickness, and WCA of both membranes (Table 1), we suggest that the higher permeability for CuONPs@CF is due to the hydrophobic character of fluorinate capped nanoparticles. According to the WCA and flux results, the flux increases in the same order of increasing the WCA (CuONps < CuONPs@CH < CuONPs@CF). Therefore, membrane hydrophobicity represents the main role on performance.

Table 1 shows a comparison between the results presented here and those previously reported results in systems with similar features (CuONPs and PVDF supported in NWPET). PVDF membranes with CuONPs have been prepared and their performances in water desalinization under a VMD regime have been reported. These membranes are of an order of magnitude thinner and 20% more porous than those here reported. Nevertheless, they showed similar flux values. Although the differences in the experimental setup should be considered, it has been reported that PVDF membranes show higher flux with a VMD setup than a DCMD setup [56]. On the other hand, the PVDF membranes supported on NWPET with similar thickness but 20% lower porous shows 12 times higher flux than ours [57]. Note that in the report of Luan et al., ΔT is double that used in our work. We suggest that the low flux values obtained in this work could be due to a low gradient in temperature achieved in our experimental setup, altogether with high thermal conductivity for the membranes. Both factors are crucial in MD efficiency [58].

All the tested membranes showed 100% salt rejection as confirmed by means of conductivity measurements. Indeed, no values are given since, during the 240 min of testing, a decrease and increase in the conductivity of the permeate and feed, respectively, was observed. Since the mass of water in the whole system was constant, an increase and decrease in salt concentration at the feed and permeated reservoirs occurred, respectively. It is important to stress that, as reported elsewhere, the robustness of membrane distillation techniques depends on the release of CuONPs from the inner area of the membrane to the permeated side and that this can be discarded unless a feed leak occurs [59]. The results presented here demonstrate the feasibility of the prepared membranes for use in the desalination of water via direct contact membrane distillation.

## 4. Conclusions

Flat-sheet PVDF membranes containing naked or hydrophobically capped CuO nanoparticles were prepared here by casting a polymer solution onto non-woven polyester fabric. Naked CuONPs formed aggregates at the highest concentrations (20%), which had a direct influence on the membrane roughness and hydrophobicity. Hydrophobic CuONPs had a similar effect on membrane surface hydrophobicity as per a decrease in the surface energy. According to the SEM micrograph images shown here, the membranes prepared with CuONPs at 5% and CuONPs@CH and CuONPs@CF at 10% showed the largest finger-like pores and were considered for membrane distillation. These membranes showed an increasing flux in the following order: CuONPs < CuONPs@CH < CuONPs@CF. We will keep developing the modification of the membrane surface to improve the hydrophobicity and increase the already reported flux. After that, it will be possible to considering scaling the production of membranes.

## Figures and Tables

**Table 1:** Comparison of flux results with similar systems reported in literature.

Membrane	Thickness (μm)	T_F_–T_p_ °C	Porosity (%)	WCA (°)	*J* (kg(m^2^·h)^−1^)	Ref.
CuO-PVDF	20–30	VMD	85–90	73–90	^(a)^ 1.25, 2.5, 4.5	[39]
PVDF-NWPET	200	80.5–20	49–54	75–85	47.6	[57]
CuONPs-PVDF-NWPET	260	80–50	63.6	81.5	1.8	t.w
CuONPs@CH-PVDF-NWPET	250	80–50	63.3	88.8	2.7	t.w
CuONPs@CF-PVDF-NWPET	220	80–50	61.3	95.6	3.9	t.w

^(a)^ Flux was measured at 1.2 kPa and at T_f_ = 27.5, 40 and 60 °C, respectively. T.w.: This work.

## Supplementary Materials

The following are available online at https://www.mdpi.com/article/10.3390/nano11061497/s1, Figure S1: PTFE scaffold for preparing PVDF composite membranes. Figure S2: Cross-section SEM micrographs of prepared membranes with different types of nanoparticles at different wt % values. Figure S3: FT-IR spectra of CuONPs capping agents. Figure S4: EDS spectra of nanoparticles at concentration of 10%.
