# Supplementary material for: PVDF Composite Membranes with Hydrophobically-Capped CuONPs for Direct-Contact Membrane Distillation
Source: Nanomaterials (Basel). 2021 Jun 5;11(6):1497. doi: 10.3390/nano11061497 (PMC8227552; doi:10.3390/nano11061497)
Supplement: Supplementary file 1 [file nanomaterials-11-01497-s001.zip › nanomaterials-1219576-supplementary.pdf]

Supporting Information

PVDF Composite Membranes with Hydrophobically-Capped CuONPs for Direct-Contact Membrane Distillation

César Saldías [1], Claudio A. Terraza [2,3], Angel Leiva [1], Joachim Koschikowski [4], Daniel Winter [4], Alain Tundidor-Camba [2,3,*] and Rudy Martin-Trasanco [5,*]

[1] Department of Physical Chemistry, Faculty of Chemistry and of Pharmacy, Pontificia Universidad Católica de Chile, P.O. Box 306, Post 22, Santiago 7820436, Chile; casaldia@uc.cl (C.S.); aleivac@uc.cl (A.L.)
[2] Research Laboratory for Organic Polymers (RLOP), Faculty of Chemistry and of Pharmacy, Pontificia Universidad Católica de Chile, P.O. Box 306, Post 22, Santiago 7820436, Chile; cterraza@uc.cl (C.T.)
[3] UC Energy Research Center, Pontificia Universidad Católica de Chile, P.O. Box 306, Post 22, Santiago 7820436, Chile
[4] Fraunhofer Institute for Solar Energy Systems (ISE), 79110 Freiburg, Germany; Joachim.koschikowski@ise.fraunhofer.de (J.K.); daniel.winter@ise.fraunhofer.de (D.W.)
[5] Departamento de Química, Universidad Tecnológica Metropolitana, Las Palmeras 3360, 8940577 Santiago, Chile
* Correspondence: atundido@uc.cl (A.T.-C.); rudy.martint@utem.cl (R.M.-T.)

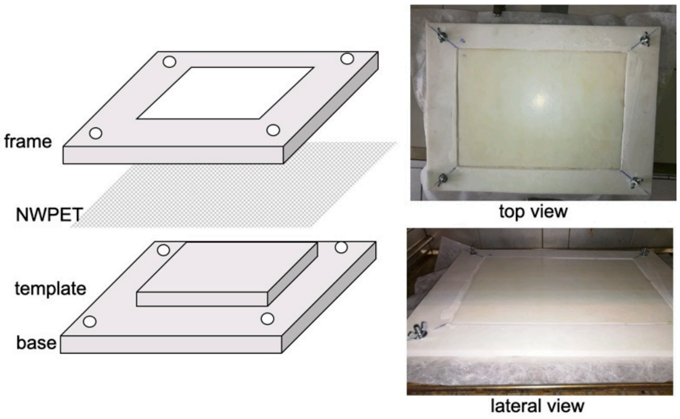

Figure S1. PTFE scaffold of 210 mm × 297 mm, for preparing PVDF composite membranes. Right: montage set-up of the hand-made PTFE device to prepare the membranes. Left: top and lateral view of prepared membranes.

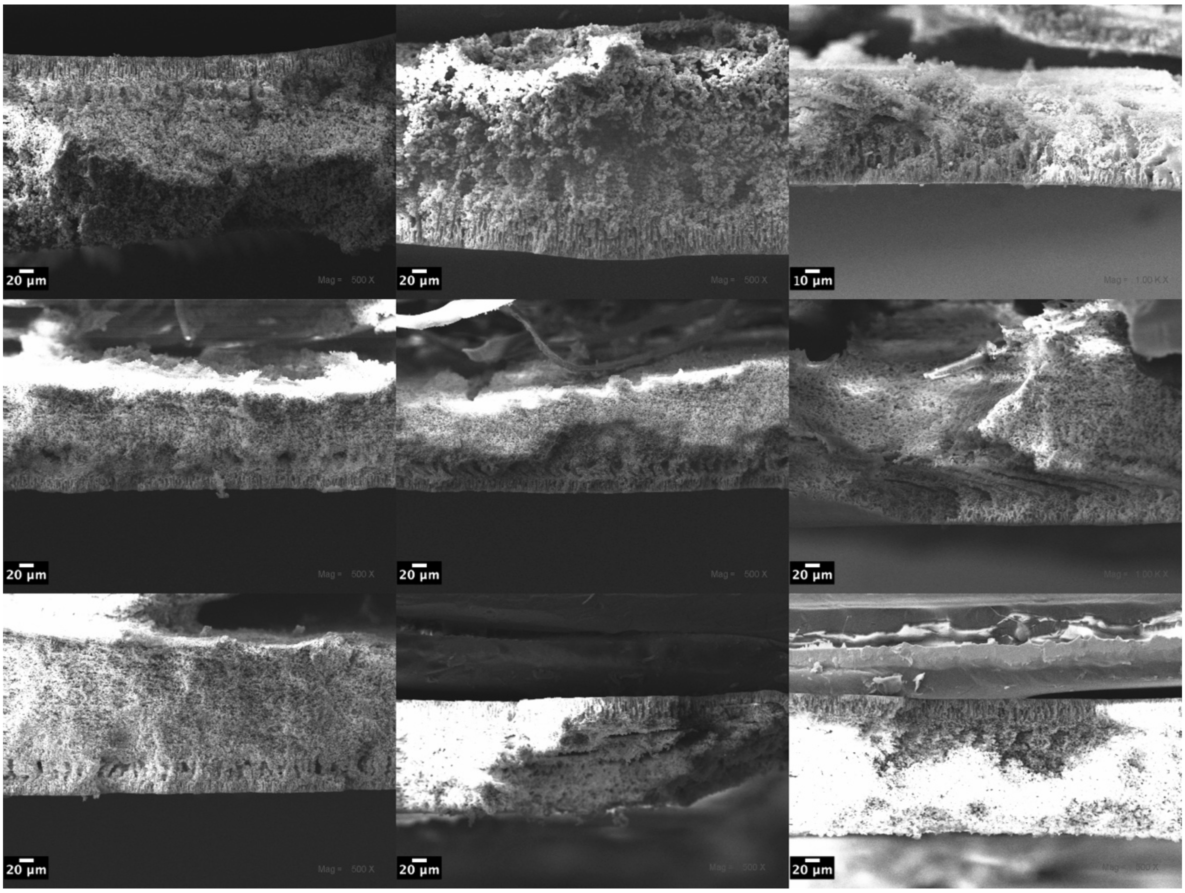

Figure S2. Cross-section SEM micrographs of prepared membranes with different types of nanoparticles at different wt%. First row: CuONPs; second row: CuONPs@CH; third row CuONPs@CF. Nanoparticle concentration, from left to right, 2%, 5% and 10%. Thickness= 240 ±40 µm.

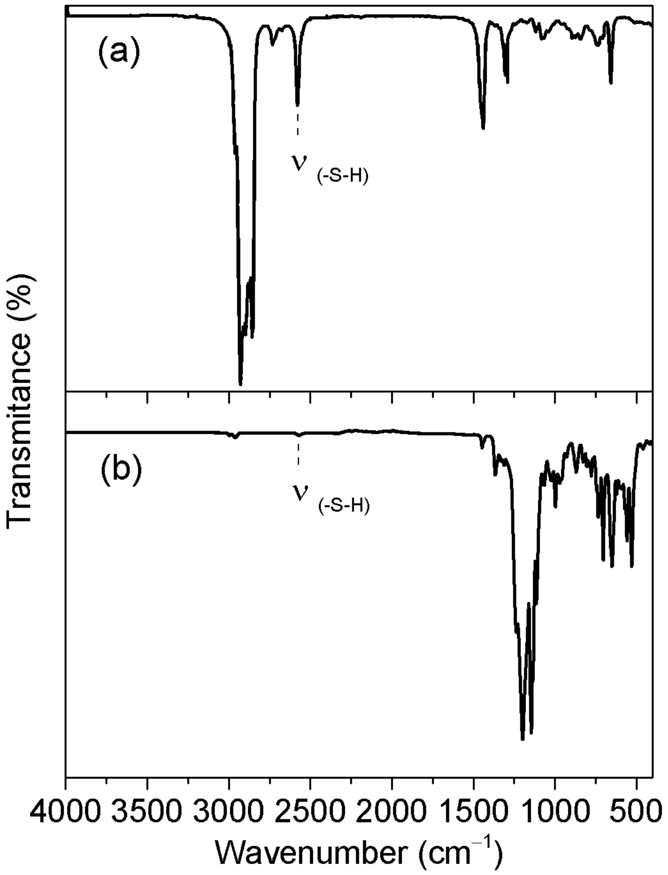

Figure S3. FT-IR spectra of (**a**) n-octanethiol and (**b**) 1H,1H,2H,2H-perfluorodecanethiol.

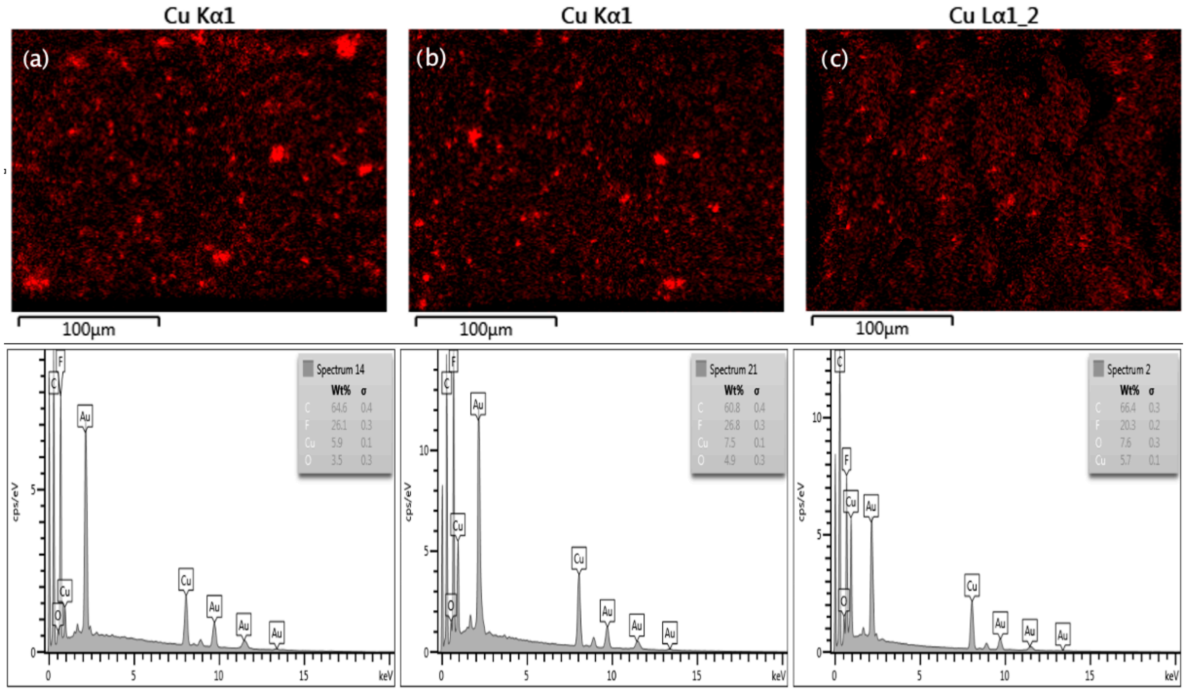

Figure S4. EDS spectrum of nanoparticles at concentration of 10% CuONPs (**a**), CuONPs@CH (**b**) and CuONPs@CF (**c**).
